# Detection and Recognition of Pollen Grains in Multilabel Microscopic Images

**DOI:** 10.3390/s22072690

**Published:** 2022-03-31

**Authors:** Elżbieta Kubera, Agnieszka Kubik-Komar, Paweł Kurasiński, Krystyna Piotrowska-Weryszko, Magdalena Skrzypiec

**Affiliations:** 1 Department of Applied Mathematics and Computer Science, University of Life Sciences in Lublin, Głęboka 28, 20-950 Lublin, Poland; elzbieta.kubera@up.lublin.pl (E.K.); pawel.kurasinski@up.lublin.pl (P.K.); 2 Department of Botany and Plant Physiology, University of Life Sciences in Lublin, Akademicka 15, 20-950 Lublin, Poland; krystyna.piotrowska@up.lublin.pl; 3 Institute of Mathematics, Maria Curie-Sklodowska University, pl. Marii Curie-Skłodowskiej 1, 20-031 Lublin, Poland; magdalena.skrzypiec@umcs.pl

**Keywords:** pollen monitoring, object detection, deep neural networks

## Abstract

Analysis of pollen material obtained from the Hirst-type apparatus, which is a tedious and labor-intensive process, is usually performed by hand under a microscope by specialists in palynology. This research evaluated the automatic analysis of pollen material performed based on digital microscopic photos. A deep neural network called YOLO was used to analyze microscopic images containing the reference grains of three taxa typical of Central and Eastern Europe. YOLO networks perform recognition and detection; hence, there is no need to segment the image before classification. The obtained results were compared to other deep learning object detection methods, i.e., Faster R-CNN and RetinaNet. YOLO outperformed the other methods, as it gave the mean average precision (mAP@.5:.95) between 86.8% and 92.4% for the test sets included in the study. Among the difficulties related to the correct classification of the research material, the following should be noted: significant similarities of the grains of the analyzed taxa, the possibility of their simultaneous occurrence in one image, and mutual overlapping of objects.

## 1. Introduction

According to the White Book on Allergy, approximately 30–40% of the world’s population suffer from an allergic disease, and the incidence of this illness is constantly increasing [1]. One of the main causes of allergies is the pollen grains of wind-pollinated plants. The presence of pollen grains in the air is seasonal and related to the flowering time. Pollen seasons are highly variable in individual years, especially for trees blooming in early spring, when the weather conditions are unstable. This entails the necessity of constant local monitoring of pollen, which provides relevant information to allergists and their patients about the risk of allergenic pollen. 

The standard method for pollen monitoring is the Hirst design volumetric spore trap [2]. Currently, two brands of this equipment are available on the market—Lanzoni s.r.l. (Bologna) from Italy and Burkard Manufacturing Co. Ltd. (Rickmansworth) from Great Britain. Samplers work continuously and prepare hourly and daily data [3,4]. They have a drum moving at 2 mm/h inside the trap. A transparent adhesive-coated tape is wound around the drum [4]. The air with bioaerosol particles is sucked in through a narrow orifice and then directed to the sticky surface. The airflow is 10 L/min, which is a value close to the volume of air inhaled by an adult in the process of breathing. The tape is replaced exactly at the same time after a week’s exposure. Then, in the laboratory, the tape with the glued biological material is cut into sections corresponding to 24 h, thus providing the basis for microscopic preparations. The qualitative and quantitative analysis of the deposited pollen grains is performed using a light microscope, most often at a magnification of 400× [5,6,7]. A lower magnification is also acceptable when the researcher is able to see details of the grain structure and perform proper identification. Regardless of the magnification used, it is required that at least 10% of the sample is analyzed [8,9]. The unit used for the expression of the daily pollen concentration is the number of pollen grains per 1 m^3^ of air [3,4]. Palynological analysis of microscopic slides is a very time-consuming process, especially during pollen release by trees, which release massive amounts of pollen into the atmosphere. For example, the daily concentration of birch pollen in the air may be as high as 12,832 pollen grains/m^3^ [10]. The development of even one preparation often requires several hours of palynologist’s work. Therefore, there is a need to facilitate and automate this process.

In this study, pollen grains with great clinical importance in allergology (*Betula*—birch, *Corylus*—hazel, *Alnus*—alder) were selected. Typically, the allergy to birch pollen is also correlated with an allergy to *Corylus* and *Alnus* pollen grains, as there is a well-documented cross-reactivity between the allergens of these plants. In spring, *Corylus* and *Alnus* pollen grains appear in the air earlier than those of *Betula*, but they can be registered simultaneously, as their pollen seasons partially overlap.

This study extends the research presented in [11], in which image classification was performed. This task involves defining target classes and training a model to recognize them. The neural network output contains class probabilities, almost equal to one for the classes detected in the image and close to zero for the other classes. The database used in [11] consisted of images containing exactly one object belonging to one class. 

In this paper, we consider images containing several objects. Our aim is to detect, classify, and count all objects. Object detection assigns the class probability and the bounding box to the object, which indicates the position of the pollen grain within the image.

YOLO is a state-of-the-art object detection algorithm, which has become an almost standard method of detecting objects in the field of computer vision. Previously, sliding window object detection was used, but then faster versions were invented, e.g., R-CNN [12], Fast R-CNN [13], and Faster R-CNN [14]. Before YOLO was developed, the networks needed to analyze images multiple times to define the regions of interest (ROI) and then classify the objects within the ROIs. In YOLO, the localization and classification are performed simultaneously, and the network reads the image only once. This explains the name of the method, YOLO—You Only Look Once. This kind of network is very fast and can be applied to detect and recognize objects in real-time systems, such as video streams.

The YOLO network has recently been applied for the detection and classification of objects in microscopic images. The second version was used in [15] among other deep learning tools for developed ZeroCostDL4Mic—an entry-level, cloud-based DL deployment platform that simplifies the procedure of the development of deep learning models for such tasks as microscopy image segmentation and object detection. The authors of [16] applied and compared four deep learning networks (Faster R-CNN, YOLOv2, YOLOv3, and RetinaNet) for the classification and localization of cells in multiple challenging 2D as well as 3D fluorescence microscopy datasets. They found YOLOv2 to be an excellent choice because it was the fastest and most accurate cell detector. In [17], YOLO was used to detect stomata in leaves of plants of several species (different varieties of common beans, barley, and soybeans). Moreover, to facilitate the use of the model, the authors developed LabelStoma, i.e., an open-source and simple-to-use graphical user interface. This tool provides a simple method for the adaptation of the YOLO model to the user’s images. The second version of this algorithm was also used in [18] for detecting and classifying white blood cells in leukemia without any traditional segmentation or preprocessing in microscopic images. The application of YOLOv3 for the recognition of pollen grains can be found in [19]. In this study, the dataset comprises sequential microscopic pollen images in the form of video recordings containing 16 pollen types with around 35,000 microscopic images per type. This set covers pollen from trees and grasses sampled in Graz (Austria) using a novel cyclone-based particle collector [20].

In our research, YOLOv5 was applied for the detection of pollen grains of three taxa (*Alnus*, *Betula*, and *Corylus*) in microscopic images. These strongly allergenic taxa are common in Central and Eastern Europe. The results from models obtained on the basis of two YOLOv5 releases, as well as different dataset variants, were compared. To eliminate bias in the model training process, we created our dataset from reference images, which contained grains of only one taxon. This solution allowed us to limit the workload of the palynologist. The detection results indicate the high accuracy of the generated models.

## 2. Materials and Methods

The most frequent species in Poland were selected for this research: *Betula verrucosa* Ehrh (syn. *B. pendula* Roth), *Corylus avellana* L., and *Alnus glutinosa* (L.) Gaertn. [21]. Reference materials were prepared by spreading pollen grains directly from the catkin onto a microscope slide. The preparations were then closed with glycerinated gelatin.

Slides with pollen grains were analyzed using an Eclipse E400 light microscope (Nikon, Tokyo, Japan) at a magnification of 600×. Photographs of pollen grains were taken using the HDCE-x5 microscope camera.

### 2.1. Dataset Preparation

**Figure 1:**
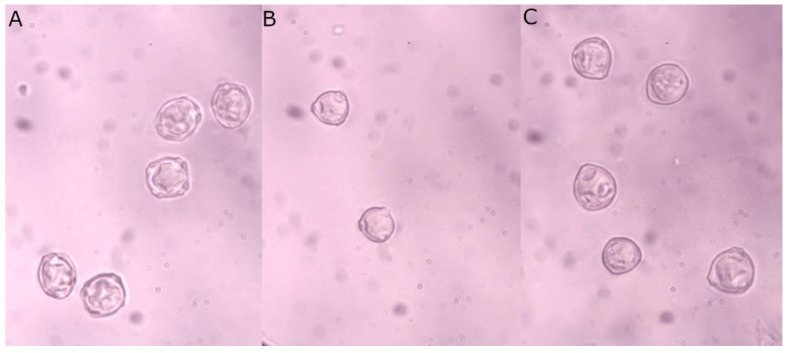
Examples of microscopic images of (**A**) *Alnus*, (**B**) *Betula*, and (**C**) *Corylus* pollen grains.

The available microscopic image database was split into a training set containing 265 images, a validation set of 114 photos, and a test set with 49 samples. Examples of photos used for training (for each taxon) are presented in Figure 1. Sixteen images with too many overlapping objects and many defocused grains were excluded. They were taken for an additional test to check the quality of the obtained models in the case of challenging pictures to be recognized.

### 2.2. Data Preprocessing and Labeling

The annotated database of the microscopic photos of pollen grains was prepared following relevant guidelines [22], and made publicly available as ABCPollenOD. The microscopic images were cropped to show only the whole grains without grain fragments. Object labeling for detection consists of marking the object’s location using a bounding box and marking the class it belongs to. The dataset was annotated twice: (1) all pollen grains belonging to the classes related to the studied taxa (DBAll) were labeled; (2) only pollen grains identified by a palynologist (DBVisible) without any doubts were marked. All co-authors verified the annotations to provide reliable ground-truth bounding boxes.

### 2.3. Investigated Models

YOLO is a general-purpose detector with an ability to detect a variety of objects simultaneously. It divides the image into a grid of cells and predicts bounding boxes, confidence for these boxes, and class probabilities for each cell. In [23], Redmon et al. introduced the first version of the YOLO technique, in which each grid cell predicts only two boxes and has one class. The second version of YOLO was introduced in [24]. A new classification model—Darknet-19—is here used as the base. YOLOv2 is better and faster than its predecessor. The main improvement is the use of the concept of anchor boxes. In 2018, Redmon and Farhadi introduced the third version of YOLO in their paper [25]. This version is more accurate than the earlier ones but slightly larger. YOLOv3 was the last version created by Redmon. It consisted of 75 convolutional layers without fully connected or pooling layers, which significantly reduced the model size and weight. Darknet-53 was used this time as a backbone architecture.

In 2020, three versions of YOLO were developed by different creators: YOLOv4 by Bochkovskiy et al. presented in [26], YOLOv5 released in 2020 by Jocher and Ultralytics Company [27] as a PyTorch implementation of YOLOv3, and PP-YOLO created by Long et al. [28]. YOLOv4 has the same backbone as YOLOv3 but introduces the concepts of the bag of freebies and the bag of specials. The bag of freebies contains, among others, some data augmentation techniques, such as Cutmix (cut and mix multiple images), MixUp (random mixing of images), and Mosaic data augmentation. An example of the bag of specials is the non-max suppression (NMS), which reduces false boxes in the case of multiple bounding boxes predicted for grouped objects. In YOLOv5, some technical changes have been made, e.g., better data augmentation, improvement of loss calculations, and autolearning of anchor boxes. The Mosaic Dataloader, which is a new concept developed by Ultralytics and first featured in YOLOv4, is used for model training. The models of YOLOv5 pretrained on the COCO database are freely available in several releases. PP-YOLO is an object detector based on YOLOv3 and PaddlePaddle [29], where ResNet [30] was used as a backbone and data augmentation was achieved by MixUp.

**Figure 2:**
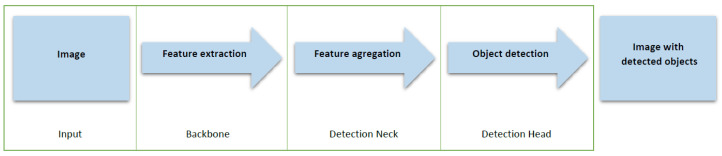
Object detector is composed of input, backbone, neck, and head parts.

In the structure of the YOLO network, only convolutional layers are used, which makes it a fully convolutional neural network. The main parts of YOLO are presented in Figure 2. The input image features are compressed through a feature extractor (backbone). The detection neck is a feature aggregator that combines and mixes features formed in the backbone and then forwards them to the detection head [31].

Data augmentation methods help to improve the model accuracy. In YOLOv5, the methods that cut and mix images containing objects of different classes produce multi-class examples, even if the input database consists of single-class samples. Therefore, YOLO with data augmentation is an appropriate method to handle microscopic images taken from reference biological material. Here, no specialist needs to be involved in the annotation process.

Two releases of YOLOv5 were chosen: YOLOv5s (small) and YOLOv5l (large). They differ in the number of layers and parameters and in the values of the initial hyperparameters. Both of these networks were pre-trained in 300 epochs on the COCO dataset [32] and are freely available.

For each release, the following four models were built by fine-tuning the initial models and taking into consideration two types of database labeling:

(1) ModelVis—trained and validated on the DBVisible set;
(2) ModelAll—trained and validated on the DBAll set;
(3) ModelAllVis—trained on DBAll and validated on DBVisible;
(4) ModelVisAll—trained on DBVisible and validated on DBAll.

### 2.4. Training Procedure

The training of the investigated models was performed in 500 epochs at the most. The model training was stopped earlier in several cases, as no improvement was observed in the last 100 epochs. The following performance measures were calculated to evaluate the model in each training epoch: precision, recall, mAP@.5, and mAP@.5:.95. 

The precision is a set-based evaluation metric. It is the ratio of true positives to the total number of objects assigned by the system to the target class. It is calculated for each class separately based on true and predicted values. The recall is the proportion of correctly classified instances of the target class to the total number of objects belonging to this class in the evaluation set for each class. The precision–recall curve can be plotted to visualize both these measures. The average precision (AP) is calculated as an area under this curve for each class separately.

In the Pascal VOC object detection challenge [31], the mean average precision measure is used for model evaluation purposes. This metric takes into consideration the fitness of the predicted bounding boxes to the actual localizations of objects. The fitness measure is defined as intersection over union (IoU). The mAP@.5 measure is the mean average precision over all the classes. @.5 means that only correctly predicted objects in bounding boxes with IoU above 0.5 are taken as positives.

In the COCO challenge, which consists of the best detection of all objects in the COCO dataset, the mAP@.5:.95 is the primary evaluation metric. It uses ten equally spaced IoU threshold values: from 0.5 to 0.95, with a step of 0.05.

The evaluation of our models was based on the fitness measure, which is a weighted mean value of mAP@.5:.95 and mAP@.5 (with weights 0.9 and 0.1, respectively) [31]. The entire training procedure was repeated three times to compare the stability of the results. The final results were presented as an arithmetic mean and standard deviation values of mAP@.5:.95 for three repetitions. 

### 2.5. Test Datasets

Besides the test set derived from DBAll and DBVisible (testAll and test Visible, respectively), testing was also done on the testDiff, which contains images excluded previously from the database due to the large number of overlapping and blurred objects. Additionally, the testMix set was prepared, with 25 images created from parts of original photos from the test set, to achieve sample images with grains of various taxa. This dataset allowed us to check whether models learned on one-class examples from reference materials can recognize objects in multi-labeled pictures. 

### 2.6. Other Detection Networks Used for Comparison

The Faster R-CNN and RetinaNet deep neural networks were applied for comparison of the YOLO results. These detectors were trained and tested on the DBAll set. We chose only one model with the highest mAP@.5:.95 values to check whether the results of these two networks could exceed the YOLO results.

#### 2.6.1. RetinaNet

The one-stage RetinaNet network architecture uses a Feature Pyramid Network (FPN) backbone on top of a feedforward ResNet architecture to generate a multi-scale convolutional feature pyramid. RetinaNet attaches two subnetworks: one for classifying anchor boxes and one for regressing from anchor boxes to ground-truth object boxes. The focal loss function enables RetinaNet to achieve accuracy at the level of two-stage detectors such as Faster R-CNN with FPN while running at faster speeds [33].

#### 2.6.2. Faster R-CNN

Faster R-CNN (region-based convolutional neural network) was the first network to combine features for region proposal with object classification [14]. It is composed of two modules. The first module is a deep, fully convolutional network that proposes regions, and the second module is the Fast R-CNN detector [13]. 

Although YOLOv2 gives comparatively low recall and more localization errors than Faster R-CNN [24], we decided to apply the YOLO network in our task due to the following advantages: fewer background mistakes than in Fast R-CNN [23] and understanding generalized object representation [24]. Moreover, one the main advantages of YOLO is the high speed of the interference process, which was not very important in this task.

 

All calculations were performed using the free cloud notebook environment Google Colaboratory [34].

## 3. Results

**Figure 3:**
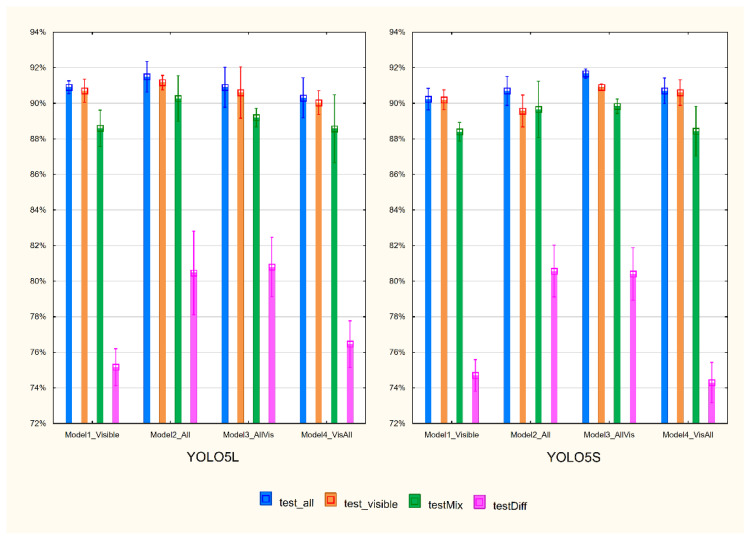
Mean and standard deviation of mAP@.5:.95 for three runs of each model evaluated on four different test datasets.

The training procedure was run three times and yielded twenty-four models. The average values and standard deviation of mAP@.5:.95 are presented in Figure 3. Very satisfactory average results of 88–92% were obtained, except for the test on test_diff with an average recognition of 74–81%. The evaluation of the 24 models on the testAll, testVisible, and testMix ranged from 86.8% to 92.4%. More than half of them reached the value of 90%. Detailed results for all models are shown in Table 1.

The comparison of the YOLOv5l and YOLOv5s models shows that both releases give similar results. The YOLOv5s models are much smaller, can be built faster, and do not require high-performance computational resources.

**Figure 4:**
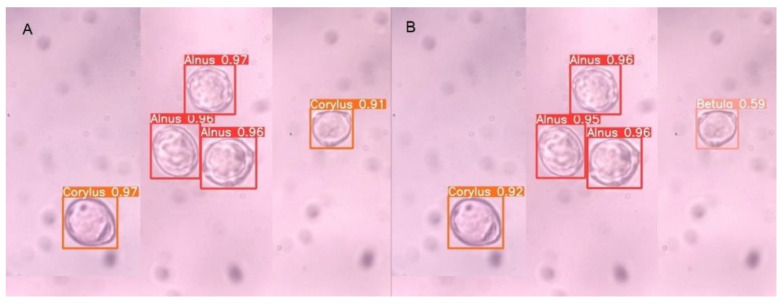
Sample image from the testMix dataset with predicted bounding boxes: (**A**) ModelAll (YOLOv5s) with Betula grains incorrectly detected as *Corylus* grain; (**B**) ModelVisAll (YOLOv5s) with a correctly detected *Betula* grain.

**Figure 5:**
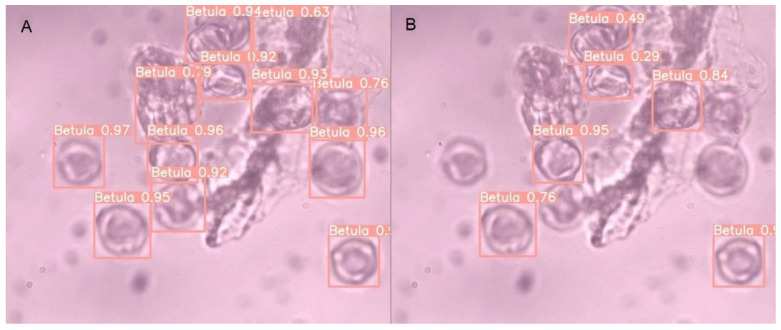
Sample image from the testDiff dataset containing only *Betula* grains with predicted bounding boxes: (**A**) ModelAll (YOLOv5s); (**B**) ModelVisAll (YOLOv5s).

Models built on different datasets give similar results when evaluated on testAll, testVisible, and testMix. An example of an image with correct and incorrect recognition for the testMix is presented in Figure 4. As indicated by the recognition results on the testDiff set, the models trained on DBAll: ModelAll and ModelAllVis exhibit much better performance. Figure 5 presents an example of an image from this set together with detection results for two of the models built based on release YOLOv5s: ModelAll (Figure 5A) and ModelVisAll (Figure 5B). Blurred grains are ignored by ModelVisAll and correctly indicated by ModelAll. The presented example also shows that ModelAll more often indicates objects that are not pollen grains. However, it is worth noting that these images are challenging even for specialists. 

The measure mAP@.5:.95 allows validation of the classification with the assessment of the fitness of the bounding box to the detected object. In the case of monitoring, it is crucial to count the occurrences of all pollen grains of a given taxon, but their precise location is not a subject of importance. The prediction results are expressed by the performance measures described above, namely precision, recall, mAP.5, and mAP@.5:.95. The average precision and recall calculated from three repetitions separately for the testAll, testVisible, and testMix datasets are presented in Table 2. According to the model, average precision ranging from 91.7% to 97.8% was achieved, while the average recall values ranged from 89.7% to 98.9%.

Confusion matrices allow the presentation of the recognition of each class separately. In Table 3, the averaged results of all twenty-four models tested on the testMix dataset are shown. The *Alnus* and *Corylus* pollen grains are rarely incorrectly detected. In particular, *Alnus* grains were never misclassified as *Betula*. The weakest recognition accuracy was noted in the case of the birch pollen grains—nearly 10% were classified as alder and 10% were recognized as hazel.

It is easy to notice that the mAP@.5:.95 values obtained with YOLOv5 on the testAll set exceed those obtained with RetinaNet and Faster R-CNN (Table 4). The results on other test sets were similar. Therefore, we did not carry out any further tests on these detectors. 

## 4. Discussion

Various monitoring methods produce different results, and it is not easy to decide which method is the most reliable. The traditional volumetric method used in Poland assumes the analysis of certain sections of collected material under a microscope and normalization of the achieved value to the number of grains contained in 1 m^3^ of air. The quantitative analysis is affected by the error associated with the randomness of samples and the palynologist’s experience. Therefore, 100% recall is not required for the automatic recognition system, because specialists do not count the grain if they are not sure to which taxon it belongs. This is why 80% accuracy of detection from the monitored material seems to be satisfactory. Our research is based on detection from reference material; hence, we expect better results.

This study is a continuation of our previous work that attempted to create classification models based on deep learning [11]. The previous study was focused only on the identification of a taxon, which is crucial in pollen monitoring. The current research is the next step towards creating an automated taxon recognition system from material collected by Hirst-type traps.

In [20], a system for the automatic creation of a digital database [19] containing microscopic photos in the form of video sequences was proposed. The research was based on the reference material; however, the preparation of slides was different than in our study. The authors proposed a new detection system working “in the wild” with high accuracy. Our research focused on improving the monitoring system currently applied in monitoring stations in Poland based on volumetric spore traps. Therefore, the previous and new results can be compared; comparison between different locations is also possible. This is all especially important as the results from the Hirst-type traps and the automatic real-time systems are significantly but not closely correlated. This was concluded in [35] for the automatic pollen monitor (PoMo), where the correlation coefficient was in the range of 0.53–0.55.

In [19], the authors describe the creation of an image database in the form of a video sequence by recording reference material for 16 types of pollen. The main problem indicated by the authors is the grouping of pollen grains into agglomerates formed during the injection of pollen samples into their system. This is a severe problem because overlapping grains impede the detection thereof. The experimental results from their study specify the number of grains detected by the system; nevertheless, in our opinion, there is a lack of comparison with the ground truth.

## 5. Conclusions

According to the model, we have achieved average precision ranging from 91.7% to 97.8% for the YOLO detector tested with sets derived from DBAll and DBVisible. The recall average values were within the range of 89.7–98.9%. This result is highly satisfactory, especially when the pollen grains of two of the studied taxa have a similar structure. The discrimination between birch and hazel pollen grains is highly problematic, especially when some grains are out of focus. Alder pollen grains can be distinguished more easily: they have five pores, whereas birch and hazel pollen grains have three pores. Moreover, in *Alnus* grains, there are characteristic arci formed of exine with thickened bows from one porus to another. 

In this study, we achieved similar detection results for YOLOv5 models built on different training sets. In particular, the differences in mAP@.5:.95 between models trained on all grains and visible ones are minimal. This allows us to conclude that not all grains have to be annotated in the training set used to create the detector. In particular, hard-to-recognize grains do not have to be labeled. Additionally, for comparison, the best result for the other networks (Faster R-CNN and RetinaNet) was the 82% value of mAP@.5:.95, which is much lower than the result obtained from any YOLOv5 run tested on testAll. 

It is worth noting that, despite the small number of training samples, the quality of the obtained models is very satisfying. Our research shows that YOLO can recognize multi-labeled images even if there are only single-labeled examples in the training set. It is a significant simplification of the preparation of the database for the detection of pollen grains, because one can use reference material without involving a palynologist in the annotation process. We forecast that it is possible to properly recognize taxa in original monitoring samples when YOLO is trained on single-labeled reference material with the same quality of photos.

We want to check our detectors on fresh biological material derived from the Hirst-type trap in the near future. We will also consider using the dataset available in [19].

## Figures and Tables

**Table 1:** Values of mAP@.5:.95 for all investigated models.

		TestAll	TestVisible	TestMix	TestDiff
YOLOv5l	ModelVis	90.6%	90.0%	88.4%	75.5%
		91.3%	91.3%	89.7%	74.0%
		90.8%	90.8%	87.7%	76.0%
	ModelAll	92.4%	91.4%	88.8%	77.9%
		90.7%	90.7%	91.2%	82.5%
		91.4%	91.4%	90.8%	81.0%
	ModelAllVis	90.3%	89.4%	89.0%	80.1%
		90.2%	90.2%	88.8%	82.7%
		92.2%	92.2%	89.8%	79.6%
	ModelVisAll	91.6%	90.8%	90.6%	77.7%
		89.7%	89.7%	86.8%	75.1%
		89.6%	89.6%	88.3%	76.6%
YOLOv5s	ModelVis	90.3%	90.3%	89.0%	75.7%
		90.8%	90.7%	88.2%	74.4%
		89.6%	89.6%	88.0%	74.0%
	ModelAll	90.0%	88.7%	88.8%	81.2%
		90.5%	89.5%	88.7%	81.6%
		91.6%	90.5%	91.5%	78.9%
	ModelAllVis	91.7%	91.0%	89.6%	81.1%
		91.4%	90.7%	90.3%	78.7%
		91.9%	91.0%	89.6%	81.4%
	ModelVisAll	91.5%	91.4%	89.6%	73.5%
		90.1%	90.0%	86.9%	73.8%
		90.5%	90.4%	88.8%	75.6%

**Table 2:** Precision and recall averaged within three repetitions for the testAll, testVisible, and testMix datasets.

		TestAll		TestVisible		TestMix	
	Model	Precision	Recall	Precision	Recall	Precision	Recall
YOLOv5l	ModelVis	94.7%	97.2%	94.1%	97.6%	94.4%	90.0%
	ModelAll	96.0%	97.7%	95.4%	97.4%	93.9%	91.2%
	ModelAllVis	96.4%	98.7%	95.6%	98.9%	94.3%	92.9%
	ModelVisAll	94.7%	95.9%	93.9%	96.0%	91.7%	91.7%
YOLOv5s	ModelVis	96.7%	96.5%	95.6%	97.4%	92.4%	92.2%
	ModelAll	97.4%	98.1%	94.9%	98.1%	92.6%	92.5%
	ModelAllVis	97.8%	97.0%	96.0%	98.1%	92.8%	90.5%
	ModelVisAll	97.5%	97.8%	96.8%	97.1%	92.2%	89.7%
Minimum		94.7%	95.9%	93.9%	96.0%	91.7%	89.7%
Maximum		97.8%	98.7%	96.8%	98.9%	94.4%	92.9%

**Table 3:** Confusion matrix of predictions for the testMix dataset averaged for all investigated models.

		True		
		*Alnus*	*Betula*	*Corylus*
predicted	***Alnus***	99.0%	9.4%	0.5%
	***Betula***	0.0%	80.4%	2.7%
	***Corylus***	0.9%	10.2%	96.1%

**Table 4:** Comparison of selected YOLOv5, RetinaNet, and Faster R-CNN mAP@.5:.95 values of detection of pollen grains in the testAll dataset.

	YOLOv5s	YOLOv5l	RetinaNet	Faster R-CNN
Run1	90.0%	90.7%	75.5%	55.7%
Run2	90.5%	91.4%	81.1%	44.2%
Run3	91.6%	92.4%	82.0%	53.0%
average	90.7%	91.5%	79.5%	51.0%

## Data Availability

Link to the ABCPollenOD dataset: http://kzmi.up.lublin.pl/~ekubera/ABCPollenOD.zip (accessed on 28 March 2022).
